# Implications of bariatric surgery and recommendations for Pakistan

**DOI:** 10.1016/j.amsu.2022.103799

**Published:** 2022-05-20

**Authors:** Khawar Abbas, Fahad Gul, Syed Naqash Haider Kazmi

**Affiliations:** Department of Surgery, Rawalpindi Medical University, Block E Satellite Town, Rawalpindi, Pakistan

**Keywords:** Bariatric surgery, Obesity, Laparoscopic procedures, Pakistan

Obesity has been a grave public health concern for the whole world but especially for developing countries like Pakistan. Asian countries are no longer behind the western ones in this trend, with south Asian countries (Pakistan and India) being among the top Asian countries for abdominal obesity when measured by WHO revised criteria of obesity for the Asian population. Abdominal obesity is nearly an accurate indicator of cardiovascular diseases, diabetes, and hypertension, so early intervention should be done [1,2].

Bariatric surgery has emerged as a definitive treatment modality for obesity considering it reduces the mortality from obesity-related complications by 28% as compared to conventional treatment of obesity. Bariatric surgery is significantly associated with sustained weight loss, remission of type 2 diabetes mellitus, and associated complications such as dyslipidemia, hypertension, and major adverse cardiovascular events. Moreover, it reduces the mortality rate by 92%, 60%, and 52% from diabetes, cancer, and coronary heart disease respectively [1]. It was concluded in a cohort study conducted in Shifa International Hospital, Islamabad, Pakistan, that bariatric surgery is superior to new lifestyle adaptations in terms of a significant reduction in raised values of prognostic parameters like HbA1C levels, systolic blood pressure, and amelioration in glycemic control after the patients undergone weight-loss surgeries [3].

Open bariatric surgeries like gastric and small bowel resections and bypasses, specifically jejunoileal bypass, were abandoned because of increased patient suffering, perioperative risks, and postoperative morbidities like vomiting, diarrhea, dehydration, and even liver cirrhosis [4]. Reduced hospital stay, rapid recovery after the operation, and decreased postoperative adverse events such as incisional hernia and wound infection made laparoscopic procedures the first choice nowadays [5]. Talking these procedures, laparoscopic sleeve gastrectomy (LSG) and laparoscopic Roux-en-Y gastric bypass (LRYGB) are commonly performed in Pakistan. Still, LSG is better than LRYGB due to a short hospital stay and comparatively brief operation time [6]. Surgeons in IFSO countries favor laparoscopic gastric banding and laparoscopic gastric bypass (LRYGB), but LSG is becoming the favorite procedure in these countries due to its efficacy [5,7].

A national survey conducted in Pakistan showed that 27.9% of women and 22% of men are obese considering Asian specific BMI cut-off value of ≥23 kg/m^2^ (Graph 1) with prevalence being highest, 42.8%, among women aged 35–54 years [8]. Despite these alarming statistics, bariatric surgery is still in its initial stages in terms of its advancement in Pakistan. Laparoscopic procedures are being performed at a limited scale, mainly in a few large cities and private sector hospitals in Pakistan. Multiple reasons behind this poor outcome are the lack of healthcare financial support, lack of adequate surgical expertise, and absence of public awareness regarding obesity, its possible consequences, and treatment options [1,9,10].

**Graph 1:**
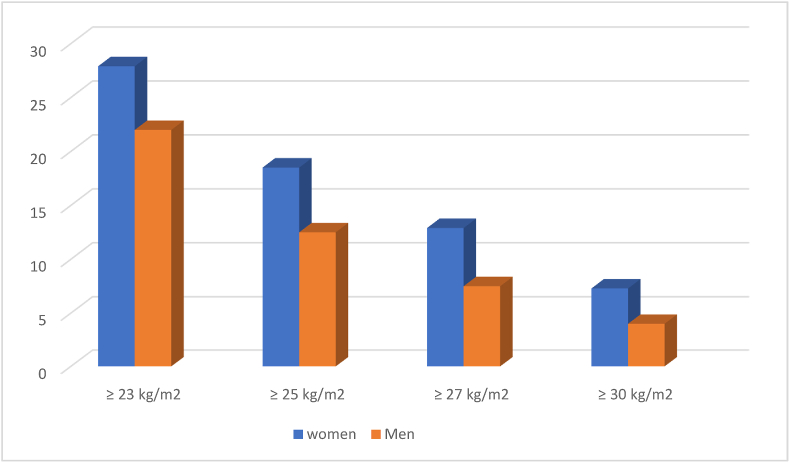
Gender specific prevalence of obesity in Pakistan.

As for recommendations, due to scarcity of data, extensive research programs should be carried out in our region to improvise guidelines to include bariatric surgery as an effective tool in the treatment of medical-resistant obesity. Bariatric surgery provides enough cushion to the healthcare system because the influx of patients to hospitals complaining about obesity and its complications-related symptoms can be decreased. Also, it has been proved to be a cost-effective cure for patients themselves as its prognosis is good and long-lived than medical therapy. Considering the deepness of the problem, public awareness drives on the national level are crucial to overcoming the illiteracy about obesity, its ill health effects, and the best treatment options that ultimately make people sensitive to seeking surgical intervention to treat obesity.

A postoperative follow-up program should be carried out for patients to access their return to normal life both physically and psychologically after the operation. Follow-up should focus on periodic clinical assessment, examination, nutrition, and physical therapy. Clinical data can be collected by doing follow-ups and can be used to determine the efficacy of bariatric surgery and its outcomes which will help establish a definite pathway for treating metabolic diseases mushrooming in Pakistan.

Funds should be granted to tertiary healthcare facilities to obtain adequate machinery and technical equipment and develop an essential infrastructure for doing laparoscopic surgeries which will lessen the burden. Most importantly, this will benefit the patients to use their regional health resources instead of going abroad for treatment. Sufficient training and fellowship programs should be initiated in multiple public and private healthcare centers to train a handsome number of surgeons countering the shortage of surgical expertise.

There is also a need to focus on future advancements in laparoscopic techniques to make them more minimally invasive procedures. Two of the techniques under study are robotic-assisted laparoscopy and the single-incision technique. There is a higher degree of accuracy in robotic surgeries. A single trocar is the only requirement in single-incision surgery, making both of them the procedures of choice in the future. Recently, a highly advanced technique introduced is natural orifice transluminal endoscopic surgery (NOTES). The procedure will be carried out by approaching the operation site from the stomach or vagina making surgeries scar-free. Besides treating obesity, these minimally invasive techniques can be an amazing choice for curing other metabolic illnesses [4].

## Ethical approval

Not applicable.

## Funding

No funding is required for the study.

## Author contributions

Khawar Abbas: Study conception, write-up, critical review, and approval of the final version.

Fahad Gul: Study conception, write-up, critical review, and approval of the final version.

Syed Naqash Haider Kazmi: Study conception, critical review, and approval of the final version.

## Consent

Not required.

## Guarantor

Khawar Abbas.

Fahad Gul.

## Registration of research studies

1. Name of the registry: Not applicable
2. Unique Identifying number or registration ID: Not applicable
3. Hyperlink to your specific registration (must be publicly accessible and will be checked): Not applicable

## Declaration of competing interest

All authors declared no conflict of interest.
